# The role of the pulmonary function laboratory in the management of hematologic diseases

**DOI:** 10.36416/1806-3756/e20240237

**Published:** 2024-11-16

**Authors:** José Alberto Neder, Denis E O’Donnell, Danilo C Berton

**Affiliations:** 1. Pulmonary Function Laboratory and Respiratory Investigation Unit, Division of Respirology, Kingston Health Science Center & Queen’s University, Kingston (ON) Canada.; 2. Unidade de Fisiologia Pulmonar, Hospital de Clínicas de Porto Alegre, Universidade Federal do Rio Grande do Sul, Porto Alegre (RS) Brasil.

## BACKGROUND

Pulmonary complications can occur in 40-60% of patients with hematologic disorders. Although acute manifestations such as infection and hemorrhage are a major concern because of their life-threatening nature, chronic respiratory complications often also increase morbidity and mortality in such patients.^1^ In this context, pulmonary function tests (PFTs) are vital for evaluating respiratory symptoms and for informing the management and follow-up of patients.

## OVERVIEW

A 52-year-old male never-smoker (Case 1) presented with complaints of progressive dyspnea and dry cough 15 months after an allogeneic hematopoietic stem cell transplantation (HSCT) for acute myeloid leukemia. In relation to the preserved pre-HSCT values, FVC and FEV_1_, expressed as percentages of the predicted value, had both decreased (to 63% and 49%, respectively). The new onset obstructive ventilatory defect (FEV_1_/FVC ratio=0.60) indicated post-HSCT bronchiolitis obliterans. This diagnosis was supported by the presence of air trapping in plethysmography (RV, 147% of predicted) and expiratory HRCT scan. A 24-year-old female never-smoker with sickle cell disease (Case 2) presented with dyspnea (with a modified Medical Research Council scale score of 3) after recurrent episodes of acute chest syndrome (new segmental opacity on chest radiograph accompanied by fever, cough, and phlegm). Spirometry revealed a proportional reduction in FVC (to 69% of predicted) and FEV_1_ (to 66% of predicted), together with a reduction in TLC (to 70% of predicted), indicative of a restrictive pattern. A mild reduction (to 70% of predicted) in DL_CO_ corrected for hemoglobin (9.6 mg/dL) and HRCT revealed chronic scarring of the lung parenchyma (pulmonary fibrosis) due to repeated episodes of acute chest syndrome with pulmonary infarction. Notably, echocardiography findings were unremarkable.

**Chart 1:**
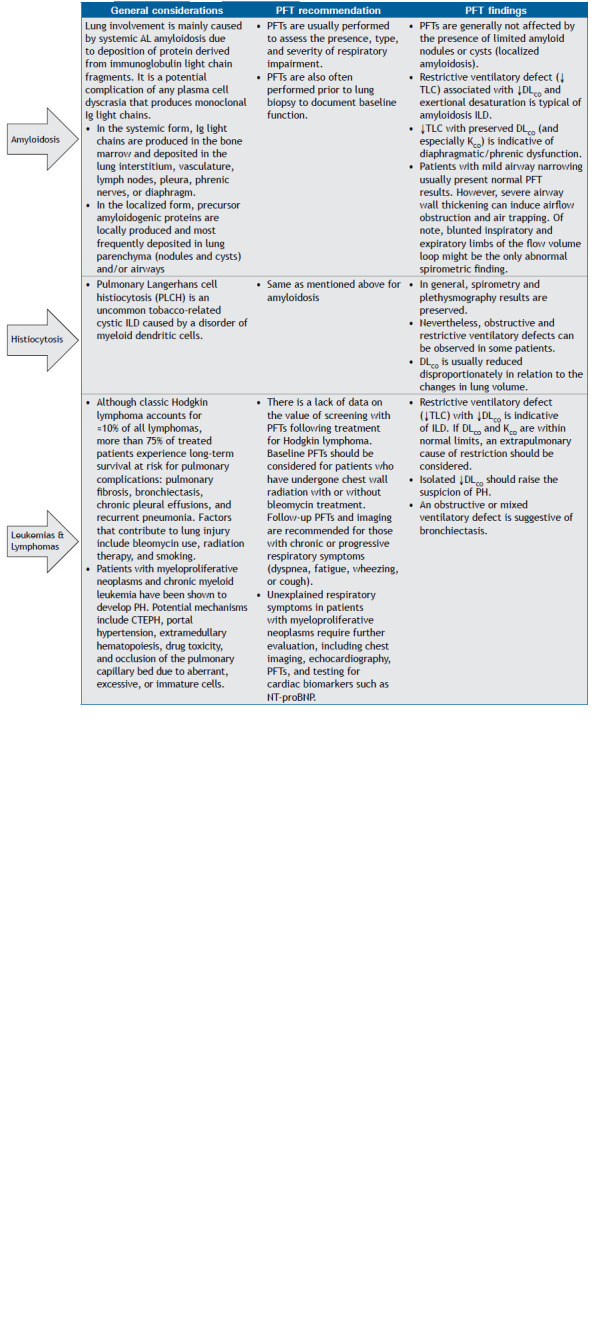
Hematologic disorders and related treatments that can cause chronic respiratory manifestations, together with general recommendations for pulmonary function testing and the main findings indicative of each respiratory complication.

With improved management leading to a reduction in infection-related mortality, noninfectious complications are becoming increasingly common after HSCT.^2^ Bronchiolitis obliterans syndrome (BOS) is a subtype of a broader category of chronic graft-versus-host disease (GVHD) involving the lungs that usually develops after the first 100 days and typically within the first 2 years after HSCT. Although BOS is characterized by the new onset of an obstructive ventilatory defect usually associated with dyspnea, cough, or wheezing, many patients are asymptomatic early in the disease process. Historically, biopsy-proven bronchiolitis obliterans was considered the only diagnostic pulmonary manifestation of chronic GVHD; that is, without the need for further testing or evidence of other organ involvement.^3^ However, because of the risks of invasive lung biopsy, PFT findings can be used as “clinical” diagnostic criteria for BOS in the right clinical context.^4^ Therefore, baseline and regular follow-up PFTs are recommended after HSCT (Chart 1). In sickle cell disease, chronic dyspnea is usually multifactorial. Common causes of such dyspnea include anemia, deconditioning, asthma, pulmonary hypertension, venous thromboembolism, and pulmonary fibrosis. Although the benefit of universal screening PFTs and echocardiography in asymptomatic individuals has yet to be determined, they should be performed in individuals with respiratory symptoms and other risk factors.^5^

## CLINICAL MESSAGE

Several hematologic diseases and their treatments can chronically affect pulmonary function by causing direct damage to the lung tissue, impairing pulmonary immune responses, or affecting pulmonary vascular function. These are diverse, heterogeneous conditions that frequently course with respiratory symptoms and complications. The use of PFTs can shed light on the underlying mechanisms and guide the management of these conditions.
